# A Review of the Mental Health Sequelae of the SARS-CoV-2 (COVID-19): Preparedness Perspective

**DOI:** 10.7759/cureus.37643

**Published:** 2023-04-16

**Authors:** Peterson Metellus, Oluwole Jegede, Colvette Brown, Danish Qureshi, Stanley Nkemjika

**Affiliations:** 1 Psychiatry, Interfaith Medical Center, Brooklyn, USA; 2 Psychiatry, Yale School of Medicine, New Haven, USA; 3 Environmental Health, Newton County Health Department, Covington, USA; 4 Population Health Sciences, Georgia State University School of Public Health, Atlanta, USA; 5 Psychiatry and Behavioral Sciences, Interfaith Medical Center, Brooklyn, USA

**Keywords:** public health, policy, covid-19, psychological, mental health

## Abstract

Despite the three significant epidemics that have rattled the world in the last two decades, many questions remain unanswered! The concept of unwanted psychological distress remains looming after any epidemic or pandemic. The public health burden of the COVID-19 pandemic still resonates with different aspects of life with predicted mental health sequelae. This review will focus on the role of natural disasters and past infectious epidemic-related mental health complications. Additionally, the study provides recommendations and policy suggestions for mitigating COVID-19-related mental health prevalence.

## Introduction and background

If history has taught us anything, we are yet to show that we have learned from it. Despite the three significant epidemics that have rattled the world in the last two decades, many questions remain unanswered! These include the uncertainty around the viral species' pathogenesis and its transmissibility mechanics. The role of urbanization [1], globalization [2], and global warming [3] in global pandemics still needs to be clarified, as is the part of social media on information spread and individual behaviors [4,5]. Hence, the reality is evidenced by the rates at which emerging infectious disease [6] gets transmitted on a large scale within days to cause global threats [7].

Following the SARS virus outbreak in 2002 (reported in China), in 2012, a new coronavirus emerged in the middle east that caused a similar symptomatic illness as SARS. Its infectivity suggested a human-to-human transmission, mainly affecting healthcare workers (HCWs) who managed infected cases. The disease was named Middle East Respiratory Syndrome (MERS) due to its documented origin and had a high fatality among immunocompromised cohorts. The MERS disease pandemic affected more than 26 countries, with 1621 confirmed cases and 584 deaths, with the maximum cases in Saudi Arabia. An association was reported between bats and coronaviruses, but the MERS-COVID-19 literature did not support any evidence of zoonotic transmission to humans through bats or any other vertebrate. Instead, considerable evidence was reported about the psychological consequence following the MERS-COVID-19 epidemic. During the first wave of the outbreak, HCWs exposed to the MERS- related tasks scored significantly higher on the Impact of Event Scale-Revised Psychosocial Stress (IES-R) instrument. This finding suggested that HCWs had a lot of psychological distress leading to increased psychiatric evaluation and care [8].

Notably, eight years after the MERS-CoV epidemic, Wuhan, one of the large metropolitan provinces in China's Hubei province, reported a cluster of patients who were admitted to a hospital with a diagnosis of 'pneumonia of uncertain etiology' now known as COVID-19. Evidence from the literature suggested that 55% of the 425 patients reported with COVID-19 in Wuhan were linked to the Huanan wholesale seafood market, which raised doubts about the alleged role of foodborne transmission [9]. History depicts that at least 167 species of bats (13% specie types) are hunted worldwide for food and other purposes [10]; So far, limited evidence shows that COVID-19 developed from bat-origin coronaviruses with a close resemblance to the SARS-CoV. Similar to the MERS-CoV, bats have been predicted as the initial source of infection, but limited evidence so far suggests a "zoonotic spill" to humans. Though the pathogenesis of this virus is still ongoing, the already known human-to-human transmission is believed to be via droplets and fomites during close unprotected contact [11]. 

Large-scale human catastrophes in the past, like the ongoing Coronavirus epidemic, have been known to result in significant mental health issues in the population affected. Considering the burden of mental health in the US before the COVID-19 pandemic, nearly one in five adults (47 million) reported having a mental illness, with over 11 million reporting a severe mental illness [12]. Hence, one wonders what the mental health prevalence might be considering the evidential impact of the pandemic so far. Still, mental health issues and substance use disorders will likely be exacerbated among people with these conditions [13]. Nationally conducted studies in the US and within the New York area following the World Trade Center (WTC) terrorism attacks reported widespread psychological distress [14,15] and some civil unrest in the form of anxiety, fear, grief, and depression [16]. Similar findings related to the sudden occurrence of environmental health threats among other populations provide further evidence of the adverse psychological impact among the affected-- the Tokyo Sarin gas attack [17], the Scud missile attacks in Israel during the Gulf War [18], and the West Nile virus outbreaks in the US [19]. The onset of psychogenic illnesses was often reported following these events [20], and even a limited Ebola virus outbreak had a significant psychological impact, making public health management difficult [14].

Facing this critical medical epidemic crisis of COVID-19, healthcare workers on the front line are involved in the day-to-day management of COVID-19 patients. Likewise, the general populace is at risk of developing psychological distress and other mental health problems. Hence, it may have contributed to the overall mental health burden among HCWs [21]. Some studies hypothesized that the incidence of psychiatric disorders in Toronto among HCWs within a year or two after a significant outbreak like the 2003 SARS would be high as the national average of psychiatric disorders in Canada [22]. However, the study's results depicted that the resilience of healthcare workers who contributed to the hospital workforce one to two years after the SARS outbreak attenuated the psychological impact with incidence rates similar to or lower than the community average psychiatric incidence [22]. Differential results were also noted for another cohort in Hong Kong. One year after the outbreak, SARS survivors still had elevated stress levels and worrying levels of psychological distress compared to the community average [23].

The fact that COVID-19 is associated with high morbidity and potential fatality may intensify the perception of personal danger. Additionally, the unforeseen shortages of medical supplies and the surge of suspected and actual cases of COVID-19 during the first wave may contribute to the pressures and concerns of HCWs. Nevertheless, it is still unclear how prepared the US and other large economies of the world are for a significant COVID-19 outbreak with its most lethal variant- the Delta variant [24], as the disease burden toll so far is not encouraging. However, despite more than 4.2 million global deaths in this review, current ongoing mathematical modeling predicts grave casualties due to the COVID-19 variant, especially for non-vaccinated people [25]. Hence, caring for those affected will challenge families and most healthcare facilities [26]. Similar to most large-scale disasters, the preparedness of healthcare systems and first-line healthcare professionals is typically an ongoing public health challenge. Hence, based on previous disaster and disease epidemic experiences from the literature regarding existing social psychological, and mental health research, we will discuss the use of this information to assist in the present COVID-19 management, future disaster planning, and crisis communication recommendations. Hence, this review is based on previous research on terrorism, the environmental health impact of major disasters, and disease outbreaks over the past two decades, as lessons can be learned from them. These events not only increase the public's awareness about environmental health concerns but will also affect the psychological well-being of local populations.

## Review

Man-made, environmental, and climate impact 

Boscarino et al. [14] found that 45% of New York City (NYC) residents following the 9/11 terrorist event were concerned about future attacks. In comparison, 20% of that number reported a fear level ranking of "10" on a 10-point analog fear scale. The study also depicted that predictors of experiencing a higher fear level during a global event included Hispanic ethnicity, lower levels of education (more severe for non-high school and high school graduates, compared to college graduates), recent stressful event, history of post-traumatic stress disorder, fear of death and reporting a high likelihood of fleeing a dangerous event [14]. Similarly, following the 2012 hurricane Sandy, 48% of New Jersey shore residents reported significant environmental health concerns, were more likely to have psychological problems in the form of post-traumatic stress disorder (PTSD) and depression, and/or to have sought mental health treatment [27]. Charlson et al.'s [28] review based on the WHO mental impact following a conflict standoff revealed that the prevalence of mental disorders in a conflict-affected population at any point in time was 22.1% [95% Uncertainty Interval (UI):18·8-25·7] and the myriads of mental disorders reported were depression, anxiety, post-traumatic stress disorder, bipolar disorder, and schizophrenia [28]. 

Pre-COVID-19: SARS, MERS, and Ebola

A study on the psychological impact of the SARS Outbreak on high-risk HCWs showed that stress levels were raised in both staffs from SARS units and healthy control subjects, using the Perceived Stress Scale (PSS). HCWs reported significantly more positive (94%, n = 256) mental disorders and more negative psychological effects (89%, n = 241) from SARS as compared to control subjects [29]. Similarly, Bai et al.'s [30] study reported that 17 staff members (5%) met the Diagnostic and Statistical Manual of Mental Disorders (DSM) IV criteria for an acute stress disorder (ASD). Furthermore, stepwise multiple logistic regression estimates from the study showed that quarantine was the most related risk factor to ASD (OR= 4.077, 95% CI=1.148- 14.48). Results also showed that 20% of the staff complained of stigmatization and rejection in their neighborhood because of their professional role in the management of SARS. Another 15% did not go home after work during the outbreak for fear of infecting their family [30]. Concerning administrative personnel, HCWs also reported forms of psychological distress like insomnia, exhaustion, and uncertainty about the continued change in the management guideline of SARS infection control procedures. Charlson et al. also concluded that the sustained high prevalence of mental health disorders in conflict-affected countries makes a compelling case for prioritizing global mental health services in conflict and post-outbreak settings [28].

Regarding MERS-CoV, mental health sequelae were noted among HCWs and families of the infected following the outbreak. Evidence from the literature documented that HCWs who performed MERS-related tasks had a higher risk for post-traumatic stress disorder symptoms even after the outbreak. The study aimed to evaluate the immediate stress and psychological impact of quarantined patients undergoing hemodialysis and university HCWs who treated patients with MERS during the outbreak. Another study by Minyoung Min on the psychological trauma of MERS victims and bereaved families reported stigmatization and discrimination even after receiving treatment and getting cleared of the disease [31]. A notable quote from an in-person interview retrieved during the study stated, "Everyone avoided us after hearing about a MERS patient in our family. Some shopkeepers threw things at us and shouted at us to leave" [31]. It is noteworthy that other mental health problems reported following MERS included chronic fatigue, depressive symptoms, and post-traumatic stress symptoms [32].

The Ebola virus epidemic was also noted to have contributed to the mental health impact among those affected, their families, and HCWs. A cross-sectional study conducted among survivors of the 2016 Ebola virus epidemic in Sierra Leone showed that symptoms of PTSD and anxiety-depression were shared at the end of the epidemic and persisted even after one year of the Ebola response [33]. Notably, the factors associated with higher reporting of any mental health symptoms included the location of residence, personal experiences during the outbreak, and perceived threat of the epidemic. Similarly, some other studies based on health professional experiences documented some cases of psychosis requiring antipsychotic medications.

COVID-19 outbreak

At the inception of this review idea, the total number of COVID-19 cases in the USA had just surpassed 1.6 million, with a total death of more than 97000, of which the state of New York makes up 30% of that population. Presently, the total number of COVID-19 cases in the US has far exceeded 41 million, and total COVID-related deaths are more than 671,000. These figures were moving targets during the pandemic. Thus far from inception, most US states ensured 'shelter in place.' This entailed closures of most businesses and schools, prohibiting any form of gatherings, requiring 14 days quarantines for travelers, continued use of face masks, and added recommendation of minimal 6-feet social distancing. Based on these measures, there was evidential research that linked social isolation [34] and quarantines [8] to mental health derangements [34]. The most common and extreme form of such sequelae is suicidal tendencies [35]. Despite the lack of literature to COVID-19 related mental health problems, Kaiser Family Foundation (KFF) tracking poll [36] conducted March 25-30, 2020, reported that 47% of those sheltering in place had mentioned adverse mental health effects due to stress related to coronavirus as compared to the 37% who were not in 'shelter.' 

Similarly, a study conducted in the US among Psychiatric HCWs between March 10- 31st, 2020, found that approximately 30% (CI: 27.4-.30.4%) of workers in the US experienced symptoms of anxiety and depression during the study period. There was also a strong association between worsening mental health and the economic consequences of the COVID-19 pandemic [37]. Supportively, since more than 65% of the US so far had filed for unemployment benefits during this pandemic, increased major depressive disorder, anxiety, distress, and higher rates of substance use disorder have been reported from previous literature [38]. Additionally, as unemployment soars and a recession loom, suicidal rates might increase, which is evidenced by the tremendous historic recession suicidal rates in the US [39]. 

The evidence for the mental health burden can also be deduced from Lai et al.'s study [40], which collated both demographic and mental health measurements from 1257 healthcare workers in 34 hospitals in China. They assessed the magnitude of mental health outcomes among HCWs managing patients exposed to COVID-19. The results of the study revealed that 71.5% of participants reported a form of psychological stress, 50.4% reported being depressed, and 44.6% of the participants confirmed anxiety disorder [40]. The health workers in Wuhan province and emergency response health workers reported a more severe mental health disorder than other HCWs. Their study also noted that female nurses were more predisposed to mental health issues as compared to male nurses, medical doctors, and other health workers, especially concerning generalized anxiety disorder (GAD) with median interquartile range (IQR) scale scores of 2.0 [0-6.0] vs. 4.0 [1.0-7.0] for men and women respectively. The estimates for the relationship between health workers outside Hubei province compared to those in Wuhan showed a lower risk of experiencing symptoms of distress with a value of (OR= 0.62; 95% CI, 0.43-0.88; P = .008). For those in the frontline of management of COVID-19 patients, they had a higher association for depressive symptoms (OR= 1.52; [95% CI= 1.11-2.09]), anxiety (OR= 1.57; [95% CI= 1.22-2.02]), insomnia (OR= 2.97; [95% CI= 1.92-4.60]), and distress (OR= 1.60; [95% CI= 1.25-2.04]). They concluded that being a woman and having an intermediate professional title level was associated with severe symptoms of depression, anxiety, and distress [40].

Similarly, another study on the progression of mental health services in China following COVID-19 [9] reported that confirmed and suspected cases of patients with COVID-19 may experience fear of severe disease consequences and contagion. The sequelae of the fear may lead to loneliness, denial, anxiety, depression, insomnia, and despair, which may lower treatment adherence for mental health patients. Cases of increased risk of aggression and suicidal tendencies were reported in China as well [9]. The prevalence of anxiety disorders was also reported to be mainly due to uncertainty about their health status and possible obsessive-compulsive behavior in the form of repeated temperature checks and sterilization practices [9]. These behavioral trends were depicted to lead to possible paranoia of being infected [9]. For mentally unstable patients in China, a total of 283 new secondary infections of COVID-19 occurred within two weeks at Wuhan Mental Health Center [9]. This astronomical number may lead to anxiety and fear of contagion within the inpatient wards. For outpatient mental health patients, the movement restrictions meant that patients may experience difficulties in receiving maintenance treatment and may end up with short-term relapse of psychological symptom conditions (e.g., hyperactivity, agitation, and self-harm). Long-term uncontrolled deterioration may lead to diminished mental and general well-being, which may degenerate to the risk of negative feelings and suicidality as a complication [9]. A similar study conducted between April 9th -24th, 2020, in New York City (NYC), which was once considered the COVID-19 epicenter in the US, assessed the mental health consequences of the COVID-19 pandemic on healthcare workers. The study's results revealed that 57% of HCWs screened positive for acute stress, 48% screened positive for depressive symptoms, and 33% screened positive for anxiety. Additionally, nurses/advanced practice providers were significantly more likely than attending physicians to screen positive for acute stress (64% vs. 40%, p < 0.001) and depressive symptoms (53% vs. 38%, p = 0.004). Nurses/advanced practice providers were also more likely than attending physicians and house staff to screen positive for anxiety (40% vs. 15% [p = 0.001] and 17% [p = 0.001], respectively) [41]. 

Synthesis or review

Even for most developed economies like the USA, the potential barriers to the development and uptake of mental health services during a national crisis like an epidemic included a lack of mental health knowledge among the health care professionals, ill-equipped diagnostic skill set, high workload for health care professionals precluding addition of mental health responsibilities, lack of drugs, the poor physical infrastructure of health, the prevalence of negative attitudes and stigma towards mental health patients, their family members and against mental health workers [42]. During the 2004 SARS outbreak, the alternate mechanism of stress for COVID-19 stems from the scarce resources needed as personal protection equipment (PPE) for the HCW, the unavailability of face masks for the general populace in America as a probable protection measure for unverified evidence of air droplet transmission modality of COVID-19 pandemic [43]. 

Based on the issue of frontline healthcare workers experiencing more severe mental health problems, healthcare institutions and government stakeholders should allocate resources towards proactive global mitigation of mental health problem surges. Hence, psychological and psychiatric management services should be amplified as soon as possible for states in the USA like New York, New Jersey, and Louisiana. This suggestion has been documented in the literature to be successful in a low-income setting; for example, a nurse-led management approach during the 2014- 2016 Ebola virus disease (EBV) outbreak within a non-specialist healthcare setting in Sierra Leone was a successful model for delivering mental health and psychosocial support services [44]

From a public health perspective, it is essential to manage psychological reactions among the public after a significant environmental threat and even before it occurs [27]. Despite this realization, most public health preparedness activities have focused on these events' technological and biomedical aspects rather than the psychological and psychiatric aspects. Following the COVID-19 health burden, mental health surveillance teams should be set up and constituted to tackle the aftermath of the pandemic. Referencing past epidemics or major national disasters in the country, public education and communication have been recommended as proactive measures to reduce or limit adverse population reactions [19]. This has been documented to provide solutions in the past of not only assuring the safety and concerns of the populace but also promoting self-protecting behaviors like stockpiling emergency supplies, building trust, and preventing the spread of misinformation [19]. 

Concerning outbreak or disaster planning considerations, it is arguable that the USA did not prepare for the pandemic on time. Hence, the media coverage and risk communications, critical to calm the populace in proactive situations, have instead incited fear across the country based on the reactive nature of the COVID-19 intervention timing. Regarding health disparities, the homeless population is densely distributed around the world's major cities, with a high disease burden. This poses challenges concerning communication, infection control, isolation, and quarantine, especially concerning the co-morbid mental health disorder unique to this population target [45].

On another note, the unique nature of novel infectious disease outbreaks is the uncertainty that arises due to the continued evolving knowledge in relation to medical science of its management [46]. This is the same for COVID-19 and past outbreaks like Ebola, SARS, and MERS, as these uncertainties breed fear among the population, transcending to different myriads of mental health problems, especially GAD, depression, PTSD, and substance use disorders. Following the 2003 SARS outbreak among HCWs, studies showed fear of contagion among HCWs, and infection of their family, friends, and colleagues. Other notable findings were feelings of uncertainty and stigmatization, reported reluctance to work, and contemplating resignation. Participants also reported experiencing high anxiety, stress, and depression symptoms, which could have long-term psychological implications. Similar mental health and psychological adjustment concerns among HCWs caring for COVID-19 patients are described among the US population.

Consequently, risk communications should incorporate this uncertainty, not ignore it, towards psychological prevention resources. This type of approach has been successful in the past, as Boscarino et al. [14] reported that worksite crisis interventions offered by NYC employers immediately after the World Trade Centre (WTC) attacks were effective in reducing the number of mental health problems (including binge drinking, depression, PTSD severity, and anxiety symptoms). Some studies proposed dispositional optimism as a preventive measure, a generally stable expectation that good things will happen [47]. These studies have linked dispositional optimism to more effective coping strategies, which leads to positive health behaviors, including a stable mental health state, leading to better health outcomes [48]. Ultimately, it will help buffer stressors, increase perceived social support, and improve health behaviors. Hence, screening for optimism to mitigate adverse mental health effects and enhance the recovery of individuals affected by COVID-19 impact is highly recommended.

Recommendations

To facilitate effective and adequate mental health services during this COVID-19 pandemic, we recommend that the knowledge and skillset gap among professionals should be bridged, especially in low-resource settings. Secondly, early engagement of concerned participants and an unconditional partnership approach with clearly defined roles and responsibilities for all parties is highly recommended to ensure authenticity and altruistic commitment to service. Thirdly, the implementation of supportive interventions geared towards improving the perceived attitude of the populace concerning mental health understanding is of utmost importance. Additionally, programs to mitigate COVID-19-related stress among HCWs should be developed, as these should integrate the preferences of HCWs [49]. For future unforeseen infectious disease pandemics, the US should consider proactive budgeting and resources to the mental health sector to prevent the burden of the disease. Finally, the US Congress should make policies incorporating mental health-related complications in planning infectious disease epidemic management.

## Conclusions

Psychosocial support may be needed for severely symptomatic patients recuperating from COVID-19 illness with possible near-death related experiences. Finally, consideration should be given to homeless people with mental illness concerning adapting the social distancing and home isolation management protocol; Hence, healthcare providers should recognize the need to communicate with the designated drop-in centers for the homeless to avoid losing patients to follow-up. In conclusion, mental health specialists should be made available while there is still a considerable burden on mental health from COVID-19 and a vast void in understanding its pathogenicity. Human and financial resources should be incorporated into the management protocol of the epidemic and the post-pandemic era because public health surveillance of mental health conditions should be an essential component of global health security efforts. Thus, mental health workers need to be aware of these developments and become more involved with preparations and management related to the psychosocial mitigation of COVID-19 impact and other public health threats in the future.
